# Changes in spinal stiffness with chronic thoracic pain: Correlation with pain and muscle activity

**DOI:** 10.1371/journal.pone.0208790

**Published:** 2018-12-11

**Authors:** Isabelle Pagé, François Nougarou, Arnaud Lardon, Martin Descarreaux

**Affiliations:** 1 Department of Anatomy, Université du Québec à Trois-Rivières, Trois-Rivières, Québec, Canada; 2 Department of Electrical and Computer Engineering, Université du Québec à Trois-Rivières, Trois-Rivières, Québec, Canada; 3 Department of Human Kinetics, Université du Québec à Trois-Rivières, Trois-Rivières, Québec, Canada; Universite de Nantes, FRANCE

## Abstract

**Objective:**

The objective was to compare thoracic spinal stiffness between healthy participants and participants with chronic thoracic pain and to explore the associations between spinal stiffness, pain and muscle activity. The reliability of spinal stiffness was also evaluated.

**Material and methods:**

Spinal stiffness was assessed from T5 to T8 using a mechanical device in 25 healthy participants and 50 participants with chronic thoracic pain (symptoms had to be reported within the evaluated region of the back). The spinal levels for which spinal stiffness was measured were standardized (i.e. T5 to T8 for all participants) to minimize between-individual variations due to the evaluation of different spinal levels. The device load and displacement data were used to calculate the global and terminal spinal stiffness coefficients at each spinal level. Immediately after each assessment, participants were asked to rate their pain intensity during the trial, while thoracic muscle activity was recorded during the load application using surface electromyography electrodes (sEMG). Within- and between-day reliability were evaluated using intraclass correlation coefficients (ICC), while the effects of chronic thoracic pain and spinal levels on spinal stiffness and sEMG activity were assessed using mixed model ANOVAs. Correlations between pain intensity, muscle activity and spinal stiffness were also computed.

**Results:**

ICC values for within- and between-day reliability of spinal stiffness ranged from 0.67 to 0.91 and from 0.60 to 0.94 (except at T5), respectively. A significant decrease in the global (*F*_1,73_ = 4.04, *p* = 0.048) and terminal (*F*_1,73_ = 4.93, *p* = 0.03) spinal stiffness was observed in participants with thoracic pain. sEMG activity was not significantly different between groups and between spinal levels. Pain intensity was only significantly and "moderately" correlated to spinal stiffness coefficients at one spinal level (-0.29≤*r*≤-0.51), while sEMG activity and spinal stiffness were not significantly correlated.

**Conclusion:**

The results suggest that spinal stiffness can be reliably assessed using a mechanical device and that this parameter is decreased in participants with chronic thoracic pain. Studies are required to determine the value of instrumented spinal stiffness assessment in the evaluation and management of patients with chronic spine-related pain.

## Introduction

Back and neck pain are very common musculoskeletal conditions in the general population, with a one-year prevalence of lumbar, thoracic and neck pain respectively estimated up to 43%, 35% and 32%.[1, 2] The prevalence rate of complementary and alternative medicine use, such as chiropractic and osteopathy, has been reported to be as high as 75% among patients with back and neck pain.[3] Spinal manipulative therapy (SMT) is widely practiced among chiropractors and osteopaths, and back pain constitutes the most frequent indication for its use.[4]

SMT is often based on the clinician’s capacity to identify mechanical changes in the spine and to select an appropriate treatment option. The identification of a "dysfunctional" spinal level that could benefit from SMT is partly based on segmental motion analysis, which can be assessed with the patient in a static position (manual segmental spinal stiffness assessment [MSSA]) or in motion (passive physiological intervertebral movement assessment [PPIVM]).[5, 6] In clinical practice, MSSA and PPIVM are assessed by contacting a spinous process with the hypothenar or thenar eminence and then applying a gradual and light posterior to anterior pressure.[5] These procedures are performed at multiple spinal levels and dysfunctional segments are usually determined based on various aspects such as the patient’s pain response, the quality of motion, the position during the movement in comparison to adjacent segments and the clinician’s experience of assessing the same spinal level in other patients.[5] Although most clinicians believe segmental motion analysis is "somewhat" or "very" accurate for estimating spinal mobility [5], conflicting and acceptable evidence have been respectively found for interobserver and intraobserver reproducibility of MSSA, while strong evidence of unacceptable reproducibility has been found for PPIVM [7, 8]. Several factors have been shown to affect the clinician’s sensations or the tissue behavior during this procedure such as patient’s anthropometrics, position and breathing as well as the procedure characteristics (load, velocity, angulation, spinal levels, contact area and breathing) [9, 10], perhaps explaining its limited reliability and inaccuracy.

To increase the validity and reliability of the assessment of spinal stiffness, mechanical devices allowing assessment standardization have been developed. These devices usually consist of an indenter, a load cell and a displacement sensor, and assess spinal stiffness by gradually applying a specific load over the targeted spinous process with a controlled velocity.[6] In addition to controlling the load, surface area, angulation and speed used during the assessment, standardized instructions related to breathing are given to the participant decreasing inter and intra individuals variability.[10] The load and displacement data recorded during the assessment are then used to calculate spinal stiffness coefficients with higher coefficients representing lower mobility. Under such conditions, the assessment of spinal stiffness reliability was shown to be "fair" to "excellent" [6, 11–13] with a recent study reporting intraclass correlation coefficients (ICC) up to 0.99 in participants with and without chronic low back pain (LBP) [14].

The assumption that SMT modulates spinal stiffness and that this latter could be used as both a diagnostic and a prognostic tool in patients with spine-related pain is at least partly supported by the current literature. Indeed, two distinct studies [15, 16] have shown an association between an immediate decrease (i.e. a return towards the mean value observed in healthy participants) in lumbar spinal stiffness following a lumbopelvic SMT and a clinically significant improvement in disability. Although these studies indicate that the assessment of spinal stiffness using mechanical devices could help identifying patients with spine-related pain that may rapidly and positively respond to SMT, only a few studies have shown a significant difference in spinal stiffness between healthy participants and participants with spine-related pain (chronic neck pain [17] or within a current episode of LBP [18, 19]). The few other studies comparing these populations did not succeed in showing a statistically significant difference between their spinal stiffness (acute, subacute or chronic LBP [14, 16, 20, 21]) and, to our knowledge, the effect of thoracic pain remains unknown.

Considering this evidence, the main objective of the present study was to compare thoracic spinal stiffness between healthy participants and participants with chronic thoracic spinal pain and, in an attempt to better understand the mechanisms by which spinal stiffness could be modulated in participants with chronic thoracic pain, to evaluate the associations between spinal stiffness and both muscle activity and pain intensity during the measurement. Furthermore, since spinal stiffness reliability has rarely been investigated in the thoracic spine, and has yet not been compared between several contiguous spinal levels, the within- and between-day reliability has also been evaluated using a subgroup of the participants.

## Material and methods

### Participants

Twenty-five healthy participants and 50 participants with chronic thoracic pain were recruited in the University community through its web portal and in the general population using a word-of-mouth strategy. The thoracic spine region was defined as the region bounded superiorly by the tip of first thoracic spinous process, inferiorly by the tip of the last thoracic spinous process, and laterally by the most lateral margins of the erector spinae muscles.[22] Inclusion and exclusion criteria are reported in Table 1 and were screened (the medical records were not accessible) at the beginning of the first session through a written check list as well as orally by the main investigator who is a chiropractor (IP). This study was approved by and carried out in accordance with the recommendations of the Université du Québec à Trois-Rivières Human Research Ethics Committee (CER-16-220-07.04). The protocol was published on protocols.io (dx.doi.org/10.17504/protocols.io.twwepfe) and written informed consent was obtained from all participants in accordance with the Declaration of Helsinki.

**Table 1:** Inclusion and exclusion criteria.

Criteria	Healthy participants	Participants with chronic thoracic pain
Inclusion criteria	18 and 60 years old. No significant thoracic pain in the past year.	18 and 60 years old. Thoracic pain for at least 3 months (constant or recurrent). Pain within T5 to T8 region indicated on the pain diagram and/or during physical examination. Quebec Back Pain Disability Questionnaire score > 0. Mean pain intensity in the past three months > 0.
Exclusion criteria	Diagnosed with a non-spine-related condition that might refers pain to the chest wall (e.g. heart, lung or oesophagus conditions). Diagnosed or suspected with one of the following conditions: spine-related inflammatory arthritis, aorta aneurism, advanced osteoporosis, neuromuscular disease, myelopathy, malignant tumors, uncontrolled hypertension, radiculopathy, neurologic deficit, thoracic herniated disc, current infection, thoracic scoliosis (Cobb’s angle > 20°). Being a pregnant woman.	

### Experiment

To assess the within- and between-day reliability of spinal stiffness assessment, all healthy participants and the first 25 participants with chronic thoracic pain took part in two identical experimental sessions that were held within 24 to 48 hours. The remaining 25 participants with chronic thoracic pain participated in the first experimental session only. At the beginning of each session, participants were asked to complete a Visual Analog Scale (VAS) for pain [23], the Quebec Back Pain Disability Questionnaire (QBPDQ) [24], the Tampa Scale of Kinesiophobia (TSK) [25] and the STarT Back Screening Tool (SBST) [26]. These questionnaires assess pain intensity (/100), disability (/100), kinesiophobia (/68 with >40 suggesting kinesiophobia) and risk of poor prognosis (/9), respectively. Following a short interview and physical examination performed by an experienced clinician, participants laid face down on a treatment table (Techniques Tables Ltd., model TT5001029, Ontario, Canada) with the arms on the arms rest for 30 minutes.

### Surface electromyography procedure

Surface electromyography (sEMG) electrodes (bipolar electrodes with a 8 mm inter-electrodes distance) were applied bilaterally at approximately 2 cm from the spine (over the belly of the thoracic erector spinae muscles and in line with muscle fibers), at the level of T5 and T8 spinous processes and between T6 and T7 spinous processes (total of 6 electrodes). Before applying the electrodes, the skin was shaved, gently abraded with fine-grade sandpaper (Red Dot Trace Prep, 3 M; St. Paul, MN, USA), and cleaned with alcohol swabs. All sEMG were recorded at 2000 Hz using Trigno Wireless EMG sensors (Delsys Inc., Natick, Massachusetts, USA), which contain two differential EMG inputs with two patented stabilizing references and therefore this system does not require an external reference electrode. Participants were then required to relax quietly on the table while sEMG activity was recorded during 4 s using EMGworks 4.2 software (Delsys Inc., Natick, Massachusetts, USA). The signal recorded by each electrode during this trial (further referred to as the sEMG normalization trial) was subsequently used to normalize the respective electrode signal obtained during the various spinal stiffness assessments.

### Spinal stiffness assessment

Following the sEMG normalization trial, spinal stiffness was assessed at T5, T6, T7 and T8 spinous processes (four times at each spinal level) and sEMG activity was concomitantly recorded during these assessments. A randomization scheme ⟨http://www.randomization.com⟩ was used to determine in which order the spinal levels would be assessed for each participant. These spinal levels were selected a priori for all participants due to technical restriction and to minimize between-individual variations due to the evaluation of different spinal levels.[10, 27]

**Fig 1:**
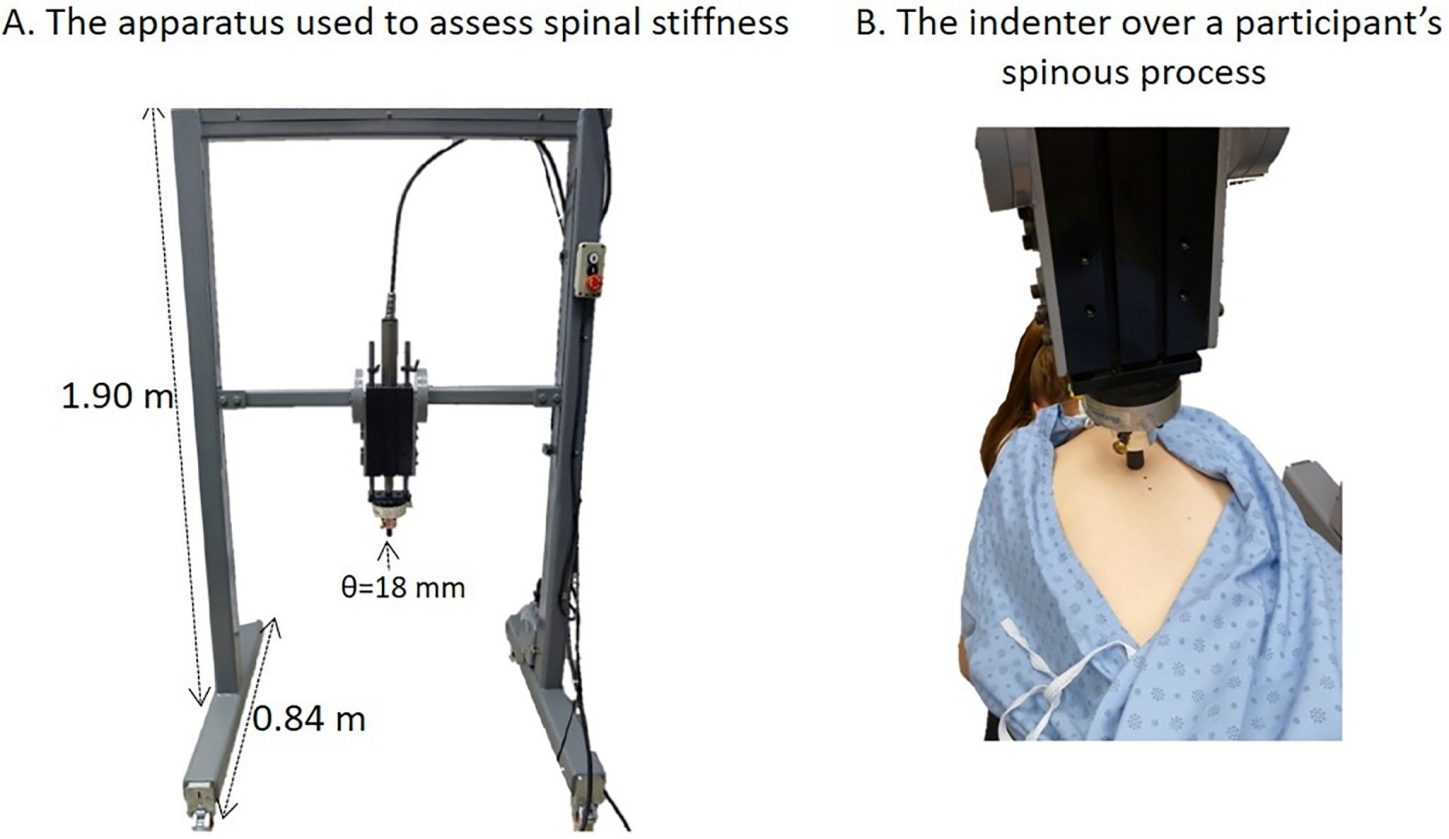
(A) Overview of the apparatus used to assess spinal stiffness with (B) a closer view of the indenter just above a participant’s spinous process. The apparatus frame has been designed to move on forward and backward as well as up and down, while the treatment table was modified to allow lateral displacements.

Spinal stiffness was measured by an apparatus using a servo-controlled linear actuator motor (Linear Motor Series P01-48x360, LinMot Inc., Zurich, Switzerland) developed to precisely generate force-time profiles (Fig 1).[28] The linear motor displaced an indenter positioned directly over a spinous process with a constant rate of force application of 18 N/s from 5 Newtons (N) to a peak force of 45 N which was maintained for 1 s before being withdrawn. Since pilot testing revealed moderate discomfort in the thoracic region for some healthy participants with a load of 60 N, the lower limit of the recommended load for spinal stiffness assessment, which is 45 N [29], was chosen. To minimize participant discomfort, a round-shaped padded rod (θ = 18 mm) was positioned at the extremity of the indenter. The researcher instructed each participant to inhale normally, then to exhale and to hold his/her breath at the end of his/her normal exhalation until completion of the spinal stiffness assessment (~5 s).[30] During exhalation, the linear motor displaced the indenter until applying a 5 N preload on the targeted spinous process. While the participant held his/her breath, a total load of 45 N was gradually applied. Each spinal stiffness assessment was separated by at least 45 s. The applied force and resulting indenter displacement were recorded using LinMot-Talk 5.1 (LinMot Inc., Elkhorn, Wisconsin, USA) at a frequency of 135 Hz.

Immediately following each spinal stiffness assessment, participants were asked to rate the mean pain intensity perceived during the trial on a 101-points VAS (0 –no pain; 100 –worst pain ever). Pain could not be rated during the assessment itself since it would have modified the lung/chest volume and therefore spinal stiffness.

## Analysis

### Surface electromyography processing

To assess the muscle activity during the assessment of spinal stiffness, the resulting bipolar sEMG signals were first digitally band-pass filtered in the frequency bandwidth 40–400 Hz (2^nd^ order Butterworth filter). A low 40 Hz cut-off allowed filtering of the electrocardiogram signal limiting contamination of the sEMG signal. The root mean square (RMS) value was computed for each electrode over a 1 s window during the assessment of spinal stiffness (between 10 and 45 N force application). The RMS value obtained for each electrode was then normalized (later referred to nRMS) to the respective RMS value calculated during the sEMG normalization trial. The mean value of the closest right and left EMG electrodes during the assessment of spinal stiffness (i.e. T5 level electrodes for T5 spinal stiffness assessment, T6-T7 level electrodes for T6 and T7 spinal stiffness assessment and T8 level electrodes for T8 spinal stiffness assessment) was then used during subsequent analyses.

### Spinal stiffness calculation

**Fig 2:**
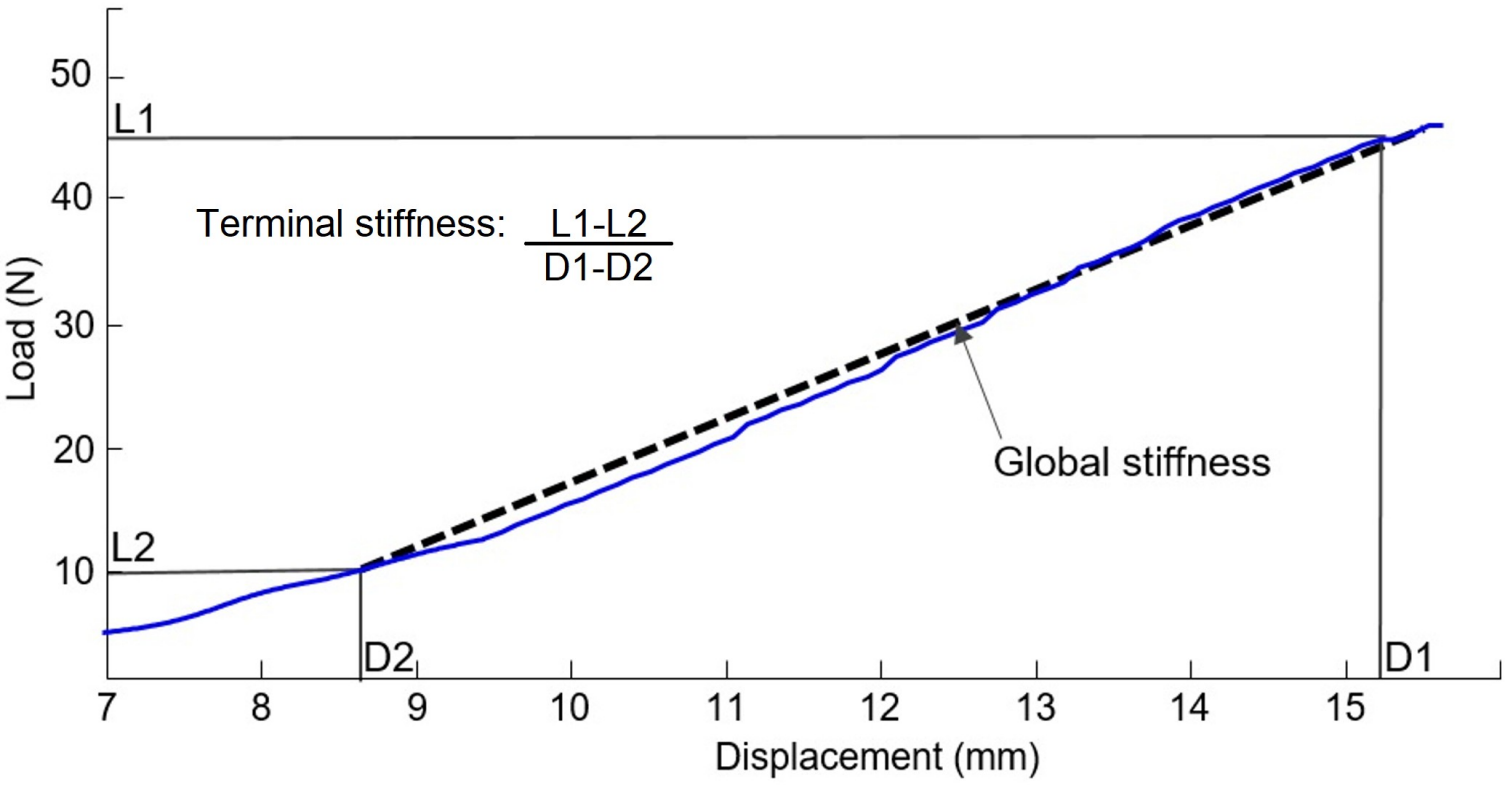
Load-displacement curve generated during a typical spinal stiffness assessment. The global stiffness was defined as the slope of the straight line (represented by the dotted line) best fitting the data between 10 and 45 N (full line) and the terminal stiffness as the ratio of the variation of the load and the variation of the displacement between 10 and 45 N ([L1-L2]/[D1-D2]).

A MATLAB (MathWorks, Natick, Massachusetts, USA) script was developed to calculate the global and the terminal spinal stiffness coefficients using the force-displacement data of each spinal stiffness assessment (Fig 2). Global stiffness was defined as the slope of the straight-line best fitting the force-displacement data between 10 and 45 N, while terminal stiffness was defined as the ratio of the variation of force and displacement between 10 and 45 N.[31] For each spinal stiffness assessment, both coefficients were computed and the average value of the second to fourth assessments of each spinal level was used in further analyses [32], excluding the within-day reliability analysis for which values per trial were used.

### Statistical analysis

#### Baseline characteristics analysis

Differences in baseline characteristics between healthy participants and participants with chronic thoracic pain (whole group and reliability subgroup) were assessed using, when appropriate, *T*-test for independent samples or Mann-Whitney U test. Differences in clinical status between the two sessions were also evaluated independently for the healthy participants and for the participants with chronic thoracic pain subset using, when appropriate, Wilcoxon’s matched pairs test or *T*-tests for dependent samples. For all analyses, statistical significance was set at *p* ≤ 0.05 and analyses were performed using STATISTICA 8 (Statsoft, Tulsa, Oklahoma, USA) and SPSS Statistics 21 (IBM, Armonk, New York, USA).

#### Spinal stiffness measurement reliability

The data from all healthy participants (n = 25) and the first 25 participants with chronic thoracic pain were used to assess spinal stiffness measurement reliability. Within- and between-day reliability was determined using ICC_3,1_ and the guideline provided by Cicchetti [33] for strength interpretation. This guideline states that an ICC value under 0.40 indicates "poor" reliability, between 0.40 and 0.59 indicates "fair", between 0.60 and 0.74 indicates "good" and over 0.74 indicates "excellent" reliability. Within-day reliability was calculated independently for both sessions considering the last three measurements per spinal level, while between-day reliability was determined using the average of these three measurements per day for each spinal level. ICCs 95% confidence intervals (95%CI) and standard error of the mean (SEM) were also computed.

#### Effect of thoracic pain on spinal stiffness, pain and muscle activity

To investigate differences in spinal stiffness between groups and between spinal levels, as well as possible interaction effects between these variables, mixed model analyses of variance (ANOVAs) were independently computed for the global and the terminal stiffness coefficients. The dependent variables (spinal stiffness coefficients) were subjected to ANOVAs comparing responses across two groups (healthy participants and participants with chronic thoracic pain) and four spinal levels (T5, T6, T7 and T8). Similarly, between-groups differences regarding muscle activity amplitude (nRMS) during assessment were determined using a 2(groups) x 4(spinal levels) mixed-model ANOVAs. Post hoc tests using Bonferroni correction were computed for significant effects. Between-group comparisons for pain intensity during assessment were performed using simple comparisons of proportions (Fisher’s exact test) and descriptive analyses. Associations between spinal stiffness and both muscle activity (nRMS) and pain intensity during assessment were explored through Pearson’s correlation or its estimated value obtained from Kendall tau rank correlation coefficient (for non-parametric data). The importance of the correlation was evaluated as being "strong" (*r* ≥ 0.70), "good" (0.50 ≤ *r* < 0.70), "moderate" (0.30 ≤ *r* < 0.50) or "poor" (*r* < 0.30).[34]

## Results

### Baseline characteristics

Participants’ baseline characteristics are presented in Table 2. Healthy participants and participants with chronic thoracic pain were similar regarding mean age, weight, height and BMI (*p* values > 0.05). Participants with chronic thoracic pain reported significantly higher score on the self-reported clinical outcomes compared to healthy participants (*p* values < 0.01). Although participants with chronic thoracic pain showed a statistically significant decrease in their QBPDQ score between the two sessions (mean difference = 1.28 [SD = 3.70], *p* = 0.01), this decrease was not clinically significant.[24]

**Table 2:** Participants’ baseline characteristics (mean and SD are presented unless otherwise indicated).

Characteristic	Healthy participants		Participants with chronic thoracic pain	Subset of participants with chronic thoracic pain	
Females: Males	12:13		26:24	12:13	
Age (years, median, LUQ†)	25 (24–31)		27 (24–36)	26 (24–32)	
Height (m)	1.72 (0.11)		1.71 (0.09)	1.72 (0.08)	
Weight (kg)	69.63 (11.24)		71.11 (14.94)	71.17 (14.54)	
Body mass index (kg/m^2^)	23.50 (2.48)		24.17 (4.07)	24.04 (4.65)	
Mean pain in the past three months (/100) *	0.00 (0.00)		30.10 (16.20)	28.56 (14.50)	
	**Session 1**	**Session 2**	**Session 1**	**Session 1**	**Session 2**
Pain at the session beginning (/100) *	0.00 (0.00)	0.00 (0.00)	20.82 (17.17)	18.56 (16.26)	18.72 (16.60)
Quebec back pain disability questionnaire (/100) * ‡	0.28 (0.79)	0.68 (2.14)	12.10 (10.03)	11.08 (9.43)	9.08 (10.41)
STarT Back Screening Tool (/9, median, range) *	0 (0–1)	0 (0–1)	2 (0–9)	1 (0–9)	1 (0–8)
Tampa scale of kinesiophobia (/68) *	23.72 (5.15)	23.20 (4.68)	29.62 (8.18)	29.72 (8.86)	28.44 (7.45)

* Statistically significant higher value in the group of participants with chronic thoracic pain compared to the group of healthy participants.

^†^ LUQ: lower and upper quartile.

^‡^ Statistically significant difference between the first and second sessions in the group of participants with chronic thoracic pain.

### Spinal stiffness measurement reliability

Reliability analyses showed ICC values suggesting an overall "good" to "excellent" within- and between-day reliability in participants with and without chronic thoracic pain in exception of T5 between-day reliability in healthy participants (Table 3). Most of the 95%CI overlapped when either groups or spinal levels were compared, suggesting that these variables do not affect reliability. However, this does not hold true for T5 between-day reliability in healthy participants for which the lower bound of the 95%CI is as low as 0.10.

**Table 3:** Intraclass correlation coefficients (ICC), with their 95%CI and SEM (N/mm) for within- and between-day reliability analyses.

Spinal level	Stiffness coefficient	Healthy participants (n = 25)						Participants with chronic thoracic pain (n = 25)					
		Within-day reliability				Between-day reliability		Within-day reliability				Between-day reliability	
		Session 1		Session 2				Session 1		Session 2			
		ICC (95% CI)	SEM	ICC (95% CI)	SEM	ICC (95% CI)	SEM	ICC (95% CI)	SEM	ICC (95% CI)	SEM	ICC (95% CI)	SEM
T5	Global	0.79 (0.64–0.89)	0.28	0.87 (0.77–0.94)	0.23	0.62 (0.14–0.83)	0.57	0.88 (0.79–0.94)	0.26	0.90 (0.82–0.95)	0.25	0.83 (0.61–0.92)	0.42
	Terminal	0.79 (0.64–0.89)	0.29	0.86 (0.75–0.93)	0.24	0.60 (0.10–0.83)	0.59	0.86 (0.74–0.93)	0.28	0.88 (0.78–0.94)	0.29	0.82 (0.60–0.92)	0.44
T6	Global	0.86 (0.75–0.93)	0.31	0.78 (0.63–0.89)	0.30	0.90 (0.78–0.96)	0.32	0.91 (0.83–0.96)	0.27	0.84 (0.72–0.92)	0.34	0.93 (0.84–0.97)	0.34
	Terminal	0.85 (0.73–0.93)	0.35	0.82 (0.69–0.91)	0.29	0.91 (0.79–0.96)	0.31	0.90 (0.82–0.95)	0.28	0.86 (0.75–0.93)	0.35	0.94 (0.87–0.97)	0.31
T7	Global	0.91 (0.84–0.96)	0.22	0.72 (0.53–0.85)	0.27	0.73 (0.38–0.88)	0.42	0.67 (0.47–0.82)	0.43	0.76 (0.59–0.88)	0.33	0.86 (0.68–0.94)	0.41
	Terminal	0.90 (0.82–0.95)	0.25	0.71 (0.52–0.85)	0.29	0.74 (0.40–0.88)	0.43	0.69 (0.50–0.84)	0.44	0.72 (0.54–0.85)	0.36	0.87 (0.71–0.94)	0.41
T8	Global	0.87 (0.77–0.94)	0.21	0.87 (0.77–0.94)	0.21	0.85 (0.65–0.93)	0.33	0.81 (0.66–0.90)	0.33	0.85 (0.73–0.92)	0.27	0.82 (0.59–0.92)	0.42
	Terminal	0.88 (0.79–0.94)	0.20	0.86 (0.75–0.93)	0.22	0.87 (0.71–0.94)	0.31	0.84 (0.72–0.92)	0.30	0.85 (0.73–0.92)	0.30	0.86 (0.67–0.94)	0.42

### Effect of chronic thoracic pain on spinal stiffness

**Fig 3:**
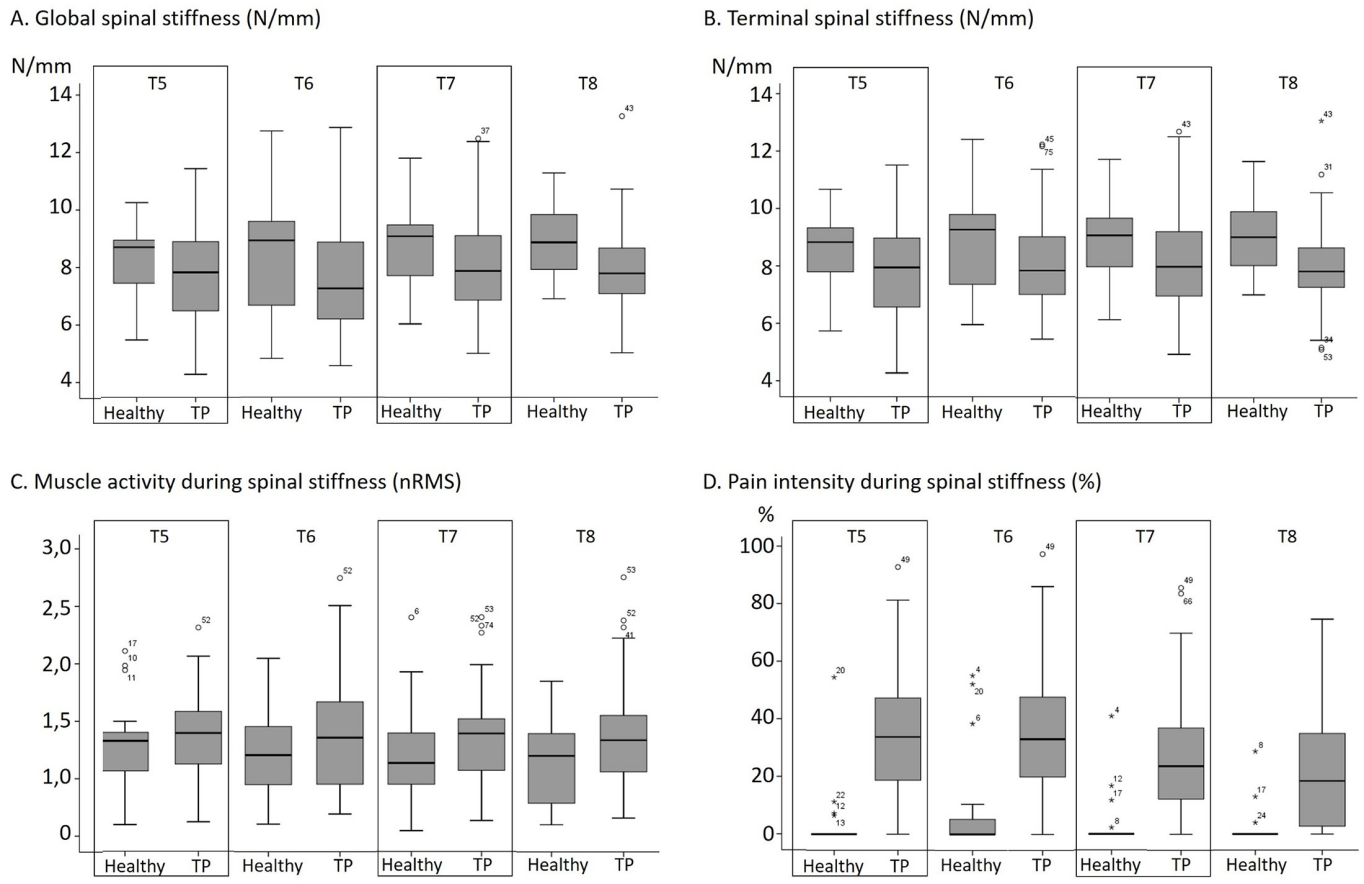
Box plots of the global (A) and terminal (B) spinal stiffness, and of both muscle activity (C) and pain intensity (D) during the assessment of spinal stiffness. Results are presented for each spinal level and independently for the healthy participants and the participants with chronic thoracic pain (TP).

A significant main effect of groups for both the global (*F*_1,73_ = 4.04, *p* = 0.048, $\eta_{p}^{2}=0.05$) and the terminal (*F*_1,73_ = 4.93, *p* = 0.03, $\eta_{p}^{2}=0.06$) spinal stiffness coefficients was observed and revealed lower spinal stiffness in presence of chronic thoracic pain. The average global and terminal spinal stiffness were respectively 8.59 ± 1.40 and 8.85 ± 1.41 N/mm in healthy participants, and 7.90 ± 1.40 and 8.08 ± 1.41 N/mm in participants with chronic thoracic pain. A significant main effect of spinal levels for the global spinal stiffness (*F*_3,219_ = 3.42, *p* = 0.02, $\eta_{p}^{2}=0.04$) was also observed with Bonferroni post-hoc test revealing that T5 (mean = 7.87 ± 1.59 N/mm) was less stiff than T7 (mean = 8.32 ± 1.70 N/mm). Such difference was not observed, however, for the terminal stiffness (*p* = 0.08). No groups by spinal levels interaction effect was observed for the two spinal stiffness coefficients (*p* > 0.05). Box plots of spinal stiffness are shown on Fig 3.

### Effect of chronic thoracic pain on muscle activity and pain intensity during assessment

Table 4 reports the correlations between spinal stiffness and both muscle activity and pain intensity evaluated during its assessment, while box plots of muscle activity and pain intensity are presented in Fig 3. Mixed-model ANOVAs for muscle activity during spinal stiffness assessment (nRMS values) showed no main effect of groups (*F*_1,72_ = 2.77, *p* = 0.10, $\eta_{p}^{2}=0.04$), spinal levels (*F*_3,216_ = 1.82, *p* = 0.15, $\eta_{p}^{2}=0.02$) or an interaction effect (*F*_3,216_ = 1.69, *p* = 0.17, $\eta_{p}^{2}=0.02$). Pearson correlations showed that muscle activity amplitude (nRMS) was not significantly correlated to the terminal and global spinal stiffness (*p* values > 0.05).

**Table 4:** Pearson correlation coefficients between spinal stiffness and both muscle activity and pain intensity during its assessment.

Factor	Group	Global spinal stiffness (N/mm)				Terminal spinal stiffness (N/mm)			
		T5	T6	T7	T8	T5	T6	T7	T8
Muscle activity (nRMS)	Healthy	-0.08	-0.03	-0.11	-0.08	-0.12	-0.01	-0.11	-0.04
	Thoracic pain	-0.04	-0.16	-0.21	-0.07	-0.04	-0.16	-0.20	-0.05
Pain intensity (/100)	Healthy	0.08†	-0.51†*	-0.57†	-0.35†	-0.06†	-0.47†*	-0.51†	-0.33†
	Thoracic pain	-0.28	-0.18	0.01	-0.16	-0.29*	-0.20	-0.04	-0.19

^†^ Pearson’s estimated value obtained from Kendall tau rank correlation coefficient (non-parametric data).

* Significant correlation at *p* < 0.05.

All participants could tolerate the 45 N load. A statistically significant higher proportion (all *p* values < 0.001) of participants with chronic thoracic pain reported pain during spinal stiffness assessment (-6.50 < z < -5.10). Indeed, 13%, 24%, 13% and 10% of healthy participants reported pain during the assessment of spinal stiffness at T5, T6, T7 and T8, while these numbers were respectively 89%, 90%, 82% and 73% in participants with chronic thoracic pain. In healthy participants, pain intensity was significantly correlated with the global (*r* value = -0.51) and the terminal (*r* value = -0.47) spinal stiffness coefficient but only at T6. In participants with chronic thoracic pain, this correlation was only significant, and of lower strength, at T5 (*r* value = -0.29) and only for the terminal spinal stiffness.

## Discussion

In the current study, thoracic spinal stiffness was compared between participants with and without chronic thoracic spinal pain, and the potential influence of pain intensity and muscle activity during assessment was evaluated to get a better understanding of the validity of spinal stiffness assessment. Although there is a widely held clinical assumption that chronic back pain is associated with an increase in spinal stiffness, the present study showed a significant decrease in spinal stiffness from T5 to T8 in participants reporting chronic thoracic pain in this spinal region. Interestingly, the highest spinal stiffness values were also observed within the group of participants with chronic thoracic pain, perhaps suggesting that both high and low spinal stiffness can be associated with chronic spine-related pain (see Fig 3A and 3B). This could explain the inconsistencies found in the current literature regarding the presence of a significant difference between healthy participants and participants with chronic neck or LBP. Indeed, few studies have shown an increase in spinal stiffness in participants with neck or LBP [17–19], while others didn’t show any significant differences [14, 16, 20, 21].

To our knowledge, the present study is the first one to concomitantly record local muscle activity and pain intensity during spinal stiffness assessment. Results showed that muscle activity amplitude was not different between both populations, and that muscle activity and spinal stiffness were not significantly correlated. Similar with observations from previous studies evaluating lumbar spinal stiffness in participants with LBP [21, 35], most participants with chronic thoracic pain reported pain during the assessment. However, pain intensity was only significantly associated with spinal stiffness at one spinal level. Interestingly, this result diverges from those reported by Latimer et al. (1996), who showed a concomitant decrease in spinal stiffness and pain intensity during the assessment of this parameter in participants with acute low back pain that reported a 80% of improvement at their second evaluation.[21] Using experimental lumbar pain in healthy participants, Wong et al. (2016) also revealed an association between pain provocation during spinal stiffness assessment, an increase in lumbar muscle activity and an increase in lumbar spinal stiffness.[35] The conflicting results between these studies and the present study highlight the possible distinctive behaviors or adaptions to pain between the lumbar and the thoracic spine or between acute and chronic pain. Until the causes of the increase or decrease in spinal stiffness with chronic back pain are better known, care should be taken when inferring results from studies investigating a spine region to another spine region or from results acquired in healthy participants to participants with chronic back pain.

With the use of a standardized protocol, an overall good to excellent within- and between-day reliability at all spinal levels (T5 to T8) was observed in both participants with and without chronic thoracic pain. To our knowledge, only one previous study aimed to compare instrumented spinal stiffness assessment reliability between participants with and without spine-related pain. Indeed, Wong et al. (2013) reported within- and between-day ICC values over 0.98 in both healthy participants and participants with chronic LBP.[14] The higher ICC values reported in Wong et al. study might arise from differences between the thoracic and the lumbar spine. Indeed, lumbar spinous processes are shorter and less angulated than thoracic spinous processes [36, 37], and perhaps small differences in the indenter location between trials in the thoracic spine might have greater impact on the measurement than in the lumbar spine. This is also supported by Edmondston et al. (1999) study, the only other study reporting reliability of thoracic spinal stiffness assessed using a mechanical device, which reported an ICC_3,1_ of 0.81 at T7 with a SEM of 1.1 N/mm for a mean spinal stiffness of 10.7 N/mm.[13] Overall, these results imply that the protocol being used in the current study is suitable for both between- and within-day comparisons in participants with or without chronic thoracic pain. Nevertheless, data showed high between-day variability in some healthy participants at T5 for the global (ICC = 0.62[0.14–0.83]) and the terminal (ICC = 0.60[0.10–0.83]) stiffness which suggests that the measurement of spinal stiffness might be less reliable in the upper thoracic spinal levels. This might be partly explained by the thoracic kyphosis. Indeed, T6 to T8 might have allowed perpendicular measurements in a greater number of participants than T5. Measurements not perpendicular to the spine could result in greater variability since the displacement of the vertebra implies a combination of movement instead of an axial translation.

### Limitations and generalisability

The minimal difference representing a clinically meaningful change in spinal stiffness has yet to be established and, therefore, it is not known if the modulation in spinal stiffness observed in the participants with chronic thoracic pain is clinically relevant. Although no relationship between muscle activity and spinal stiffness was observed in the current study, it cannot be excluded that deeper muscles, such as multifidus muscles, might have increased their myoelectric activity in response to pain during the assessment of spinal stiffness. Researchers should consider using intramuscular electromyography electrodes to evaluate the potential activation of deeper muscles. Moreover, comfort during spinal stiffness assessment should be optimized to alleviate muscle contraction due to pain during evaluation. This issue can be achieved by increasing the size of the indenter and/or modifying the indenter padding.[38] Another limitation is that the angulation of the indenter device could not be modified and therefore, some measurements might not have been done perpendicularly to the vertebra which has been reported to decrease the spinal stiffness.[2, 39] This could consequently explain the lower spinal stiffness observed at T5 (7.87 ± 1.59 N/mm) compared to T7 (8.32 ± 1.70 N/mm) ($\eta_{p}^{2}=0.04$). The results of the current study might not be generalizable to patients reporting back pain related to specific pathology such as arthritis or severe osteoarthritis. Finally, although pain or tenderness within T5 to T8 area was reported by all participants with chronic thoracic pain during the physical examination, the current study might not have targeted the main pain area in few participants with chronic thoracic pain. Future studies should consider evaluating spinal stiffness at all thoracic spinal levels.

### Clinical implications

Clinicians should be aware that perception of a variation in spinal stiffness between adjacent spinal levels or between individuals might not represent true variations and can simply be due to human factors, such as anthropometric [40, 41] and gender [41, 42], or protocol factors such assessment angulation [39, 43], speed of application and applied load [29, 44]. Based on the current study results, clinicians should not systematically look for an "hypomobile" spinal level when evaluating patients with chronic thoracic pain and should consider the possibility of increased spinal mobility.

## Conclusion

This study is the first to measure spinal stiffness at multiple thoracic spinal levels. The results demonstrated a decrease in thoracic spinal stiffness in participants with chronic thoracic pain compared to healthy participants. The data does not support the hypothesis of an association between spinal stiffness and a muscle contraction due to pain provocation during the measurement. More research is thus required to determine the value of instrumented spinal stiffness assessment in the evaluation and management of patients with chronic spine-related pain.

## Supporting information

S1 File Data necessary to replicate the analyses. (XLS)
